# The very rare association between T-cell acute lymphoblastic leukemia and down syndrome: a case report and review of the literature

**DOI:** 10.3389/fped.2026.1820038

**Published:** 2026-05-18

**Authors:** Giacomo Gotti, Laura Rachele Bettini, Stefano Rebellato, Valentino Conter, Antonella Colombini, Veronica Leoni, Alessandra Sala, Marco Spinelli, Andrea Biondi, Grazia Fazio, Adriana Cristina Balduzzi, Carmelo Rizzari

**Affiliations:** 1 Pediatrics, Fondazione IRCCS San Gerardo dei Tintori, Monza, Italy; 2 School of Medicine and Surgery, University Milano-Bicocca, Monza, Italy; 3 Tettamanti Center, Fondazione IRCCS San Gerardo dei Tintori, Monza, Italy

**Keywords:** acute lymphoblastic leukemia, down syndrome, genetic characterization, T-cell acute lymphoblastic leukemia, treatment strategy

## Abstract

**Background:**

Children with Down syndrome (DS) show a higher incidence of acute lymphoblastic leukemia (ALL) compared to general population. They are almost entirely affected by B-cell precursor ALL, linked to the constitutional chromosome 21 trisomy and associated with recurrent mutational patterns. The occurrence of T-cell ALL (T-ALL) is extremely rare in DS patients, with only few reports described. This entity remains poorly characterized either genetically and clinically, and there are no standardized guidelines for the treatment of these rare and fragile patients. Here we describe the case of a child with DS and T-ALL and provide comprehensive genetic characterization of his disease.

**Case report:**

A previously well 7-year-old boy with DS was diagnosed with T-ALL presenting with leukocytosis, mediastinal mass and central nervous system involvement (CNS2). Genetic studies showed a complex karyotype with the translocation t(1;14)(p32;q11) and deletion of chromosome 9p. RNA sequencing was negative for fusion transcript. Next-generation sequencing analysis for somatic mutations on DNA material showed pathogenic variants in *NOTCH1* and *FBXW7*. Chemotherapy was started according to AIEOP-BFM ALL 2017 study. During induction phase, the patient suffered from multiple severe complications, requiring significant treatment modifications and an anthracycline-free induction. However, the patient presented favorable response achieving minimal residual disease negativity. He is currently on maintenance therapy 15 months from the diagnosis.

**Conclusion:**

This case provides the first detailed genetic characterization of T-ALL in a child with DS. The findings of typical T-ALL somatic mutations and genetic alterations suggest a sporadic leukemogenesis origin, distinct from the specific pathway associated with B-cell precursor ALL. We confirm the rarity of this entity and the extreme susceptibility to treatment complications. An improved knowledge and characterization of DS T-ALL might be helpful to inform clinicians about treatment decision making for this very rare disease.

## Introduction

The incidence of acute lymphoblastic leukemia (ALL) in children with Down syndrome (DS) is 20–30 times higher compared to general population (1). It is almost exclusively of B-cell precursor origin (BCP-ALL), and is characterized by recurrent mutational patterns with high prevalence of CRLF2 overexpression, JAK2 mutations and RAS pathway activation (2, 3). The occurrence of T-cell ALL (T-ALL) in DS patients is exceptionally rare and remains poorly characterized either genetically and clinically (4–7). The Ponte di Legno Group has described a large international retrospective analysis of 708 children with DS-ALL treated between 1995 and 2004 and only 5 patients had T-ALL, in addition also lacking genetic characterization (4). Pediatric ALL protocols usually include recommendations for BCP-ALL DS patients, but no specific guidelines have been released for DS T-ALL.

## Case report

Here we present a 7-year-old boy with DS presenting with T-ALL with a full genetic ALL characterization. The child was admitted for persistent fever, mediastinal mass, leukocytosis (WBC count 52,600/µL with 57% of lymphoid blasts), mild anemia (12,7 g/dL) and thrombocytopenia (67,000/µL). No previous relevant medical history was reported. After the diagnosis of ALL and the start of steroid therapy, he was referred to our hospital in poor clinical condition due to tumor lysis syndrome (creatinine 0.9 mg/dL, phosphate 5.5 mg/dL, hyperkalemia 5.4 mEq/L, serum lactate dehydrogenase 2055 U/L, uric acid 15.5 mg/dL, WBC 1,710/µL (30% lymphoid blasts), hemoglobin 5.5 g/dL, platelets 1,000/µL), and bilateral pneumonia which prompted the admission to intensive care unit for respiratory support. Diagnostic bone marrow (BM) aspirate showed 75% of lymphoblasts and flow cytometric analysis showed immunophenotype of T-ALL (EGIL T-III, positive cCD3, CD7, CD45, CD99, low sCD3; negative CD10, CD19, CD33, CD34, CD117 and HLA-DR).

Genetic studies were performed on diagnostic BM sample. G-banding revealed a complex karyotype [47,XY,add(Y)(q12),t(1;14)(p32;q11),der(6)t(6;?)(q21;?),del(9)(p?22p13),+21c (20)], with the translocation involving the T-cell receptor (TCR) and the deletion of chromosome 9p commonly found in T-ALL. TCR clonality analysis identified 5 clonal rearrangements including n°2 TRB, n°2 TRG and one TRD; among them the TR Vb18 Jb2.3 and the Vg11 Jg1.3/2.3 were used for molecular minimal residual disease (ASO-PCR MRD) monitoring. Although the translocation TRA::TAL1 was not detected by RNA-sequencing for fusion transcripts (Universal RNA-Seq kit from Tecan with targeted globin transcripts depletion with AnyDeplete, sequencing on NextSeq2000 Illumina instrument), the TAL1 expression analysis in our T-ALL cohort confirmed that the test sample is one of highest among 120 T-ALL cases, as high as the most overexpressing TAL1 case (data not shown). Moreover, the patient transcriptomic profile is clustering in the TAL1 DP-like subgroup according to the Polonen et al. study (data not shown) (8). Next-generation sequencing (NGS) analysis for somatic mutations on DNA material was performed using a validated clinical NGS panel profiling 39 genes involved in leukemia pathogenesis (9) and showed a pathogenic variant in *NOTCH1* (p.Leu1678Pro) with a variant allele frequency (VAF) of 37.4% and a likely pathogenic mutation in *FBXW7* (p.Arg479Gln) genes with a VAF of 41.7%, both reported in pediatric T-ALL.

**Figure 1:**
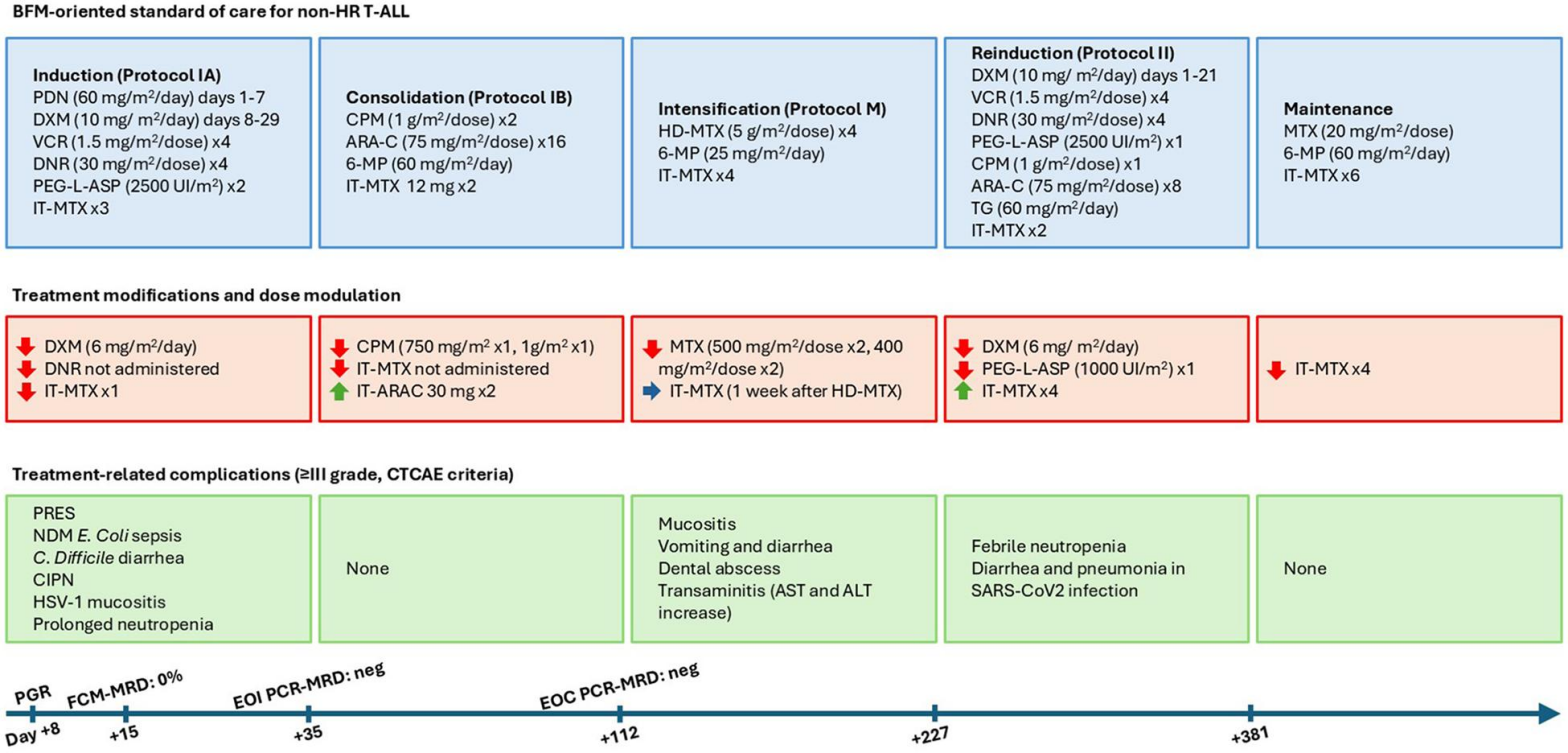
Outline of treatment phases, chemotherapy drugs and dosages according to BFM-oriented standard of care for non-HR T-ALL, modulation of therapy, toxicities and treatment-related complications and disease monitoring and response. ARA-C, cytosine arabinoside; ALT, alanine amino transferase; AST, aspartate amino transferase; CIPN, chemotherapy-induced peripheral neuropathy; CPM, cyclophosphamide; DNR, daunorubicin; DOX, doxorubicin; DXM, dexamethasone; EOC, end of consolidation; EOI, end of induction; FCM, flow-cytometry; IT, intrathecal; MRD, minimal residual disease; MTX, methotrexate; NDM, New Delhi metallo-*β*-lactamase; PEG-L-ASP, pegylated asparaginase; PCR, polymerase chain reaction; PGR, prednisone good response; PDN, prednisone; PRES, posterior reversible encephalopathy syndrome; TG, thioguanine; VCR, vincristine; 6-MP, 6-mercaptopurine.

Treatment started according to a modified AIEOP-BFM ALL 2017 study protocol (NCT03643276) with prednisone prephase, and on day +8 shifted to dexamethasone (DXM) as per protocol indications due to the favorable prednisone response. First lumbar puncture with intrathecal methotrexate (IT-MTX) performed on day +4 showed the presence of blasts but <5 leukocytes/µL (CNS2). During the induction phase (Protocol IA) the patient developed several complications. He developed posterior reversible encephalopathy (PRES) one day after the first IT-MTX with typical alterations shown by brain MRI and electroencephalogram. Surveillance rectal swab at admission was positive for New Delhi metallo-*β*-lactamase (NDM) type *Escherichia Coli* that caused fever and bloodstream infection on day +16, successfully treated with aztreonam, ceftazidime/avibactam and colistin. In addition, he suffered from *Clostridium Difficile* enteritis, grade III chemotherapy-induced peripheral neuropathy, and herpetic (HSV-1) mucositis, requiring specific pharmacological treatment and physical support therapy. Due to the patient's poor clinical conditions, the dose of DXM was reduced, anthracyclines were omitted and only one IT-MTX dose was given (at day +4). Treatment modulations and major complications for each phase of treatment are reported in Figure 1. Consolidation phase IB was given without complications. Despite the reduced induction phase intensity, the flow-cytometry assessment of BM aspirate performed on day +15 showed no blasts, and minimal residual disease (MRD) measured by polymerase chain reaction (PCR-MRD) was negative both at the end of induction (EOI, sensitivity as mean of QR = 10^−4^) and at the end of consolidation (EOC, sensitivity as mean of QR = 5 × 10^−4^), qualifying the patient as MRD-SR (10). In Protocol M MTX was given at intermediate dose (ID-MTX) (500 mg/sqm) with IT-MTX administered the week after each ID-MTX infusion and associated with leucovorin rescue. Since the patient was CNS2, additional IT-MTX was given during a modified Protocol II and maintenance therapy with 6-mercaptopurine and oral MTX. Supportive therapy included antifungal prophylaxis during intensive phases (induction Phase IA and reinduction Protocol II) with B-liposomal amphotericin B and antiepileptic therapy with levetiracetam until the interruption of intrathecal therapies. Because of the gut colonization by the NDM E. Coli, contact isolation precautions were maintained across the whole treatment period. The patient is currently on maintenance therapy 14 months after diagnosis and in persistently good clinical conditions. A summary of patient characteristics and treatment outcome is shown in Table 1.

**Table 1:** Summary of patient characteristics, genetic findings and outcome.

Characteristics	Patient
Gender	Male
Age (years)	7.1
WBC (cells/µL)	52,600
CNS status	CNS2
Mediastinal mass	Yes
Karyotype	47,XY,add(Y)(q12),t(1;14)(p32;q11), der(6)t(6;?)(q21;?),del(9)(p?22p13),+21c [20]
RNA sequencing	Negative
NGS analysis	*NOTCH1* p.Leu1678Pro (VAF 37.4%) *FBXW7* p.Arg479Gln (VAF 41.7%)
CR at EOI	Yes
Status	Alive in CCR
Survival (months)	14

CCR, continuous complete remission; CR, complete remission; CNS, central nervous system; EOI, end of induction; NGS, next-generation sequencing; VAF, variant allele frequency; WBC, white blood cell count.

## Discussion

Despite the improvement obtained over the last decades with conventional chemotherapy in pediatric ALL, the outcome of children and adolescents with DS ALL remains poorer compared to non-DS patients, mainly due to increased treatment-related toxicity requiring treatment modulation, higher incidence of adverse cytogenetic alterations, slower response and higher incidence of mortality and relapses (4, 7, 11, 12). A recent large retrospective study from the Children's Oncology Group reported an event-free survival of 79.2 ± 1.6% for DS BCP-ALL, significantly inferior to non-DS patients (87.5 ± 0.3%) in the same study period (12). The vast majority of children and adolescents with DS present with BCP-ALL while T immunophenotype as diagnosed in our patient is an exceptional occurrence in this population. Indeed, a retrospective study conducted by the Italian Association of Pediatric Hematology and Oncology (AIEOP) described only one patient with T-ALL (<1%) among 120 patients with DS and ALL enrolled on 6 consecutive trials from 1987 to 2004 (7) and none was found in the following AIEOP-BFM ALL 2009 and AIEOP-BFM ALL 2017 studies (unpublished data).

Despite the complicated and lower intensity induction treatment performed, mainly due to important clinical complications and side effects, our patient showed an excellent response achieving MRD negativity at EOI. Interestingly, four of the five patients with DS T-ALL described by The Ponte di Legno group achieved CR, whilst one patient died after presenting a very early relapse (4). However, even if long-term follow-up data was at least 5 years after diagnosis, the remaining data were quite limited in that report, without MRD response, additional genetic characterization and treatment details. Indeed, genetic studies reported were restricted to conventional karyotype, showing alterations only in one patient (complex karyotype).

DS patients are more vulnerable to treatment related toxicity and suffer from higher incidence of deaths in induction and in CR (4, 7, 11, 12). Therefore, although cases presenting with non-DS T-ALL are generally treated with 4 drugs including anthracyclines, a dose reduction of the drugs used or, alternatively, a 3-drug induction without anthracyclines could be applied in DS patients. BFM-like consolidation phase (induction phase IB) is generally well tolerated in DS patients but it might require treatment intensity modifications, require prolonged hospitalization, imply treatment delays and obligate to specific supportive therapy especially during neutropenia phases. HD-MTX cycles are highly effective to prevent CNS relapses in T-ALL and represent a fundamental part of the CNS-directed therapy of many protocols. However, given the high susceptibility to MTX-related toxicity displayed by DS patients, lower doses are generally administered in DS patients. Indeed, our patient presented significant toxicity and treatment delays, even when a reduced dose intensity was planned and delivered. The randomized COG AALL0434 study proved the superiority of the Capizzi regimen (escalating MTX plus asparaginase) compared to the standard HD-MTX approach in T-ALL patients (13); therefore, since HD-MTX cycles and intensive blocks of chemotherapy containing HD-MTX are very toxic for DS patients, the use of the Capizzi regimen and reinduction Protocol II could be adequate to either non-HR and HR DS T-ALL patients. As suggested in many ALL protocols for DS patients leucovorin rescue after systemic MTX, including IT-MTX, should be administered for longer times in respect of the strategy adopted in non-DS patients. Similarly, given the high incidence of treatment related mortality, hematopoietic cell transplantation should be restricted only to those with very poor early response, if feasible.

Nelarabine has been also used in first-line treatment for T-ALL but its efficacy is still debated (14). The addition of nelarabine improved outcome in children and young adults with intermediate and high-risk T-ALL in the COG AALL0434 study, with results particularly favorable in patients with CNS3 (15). In contrast to North American protocols, the use of nelarabine in Europe is mainly limited to relapsed/refractory diseases and is not part of the BFM approach for newly diagnosed T-ALL. Nevertheless, nelarabine may not be suitable for DS patients given the risk of severe neurotoxicity and more conventional treatment looks preferable and safer.

Recently, molecular profiling studies in T-ALL have identified a common aberrant overexpression of the anti-apoptotic protein BCL-2, therefore making suitable the use of drugs such as venetoclax in these diseases; however, the response to venetoclax, a BCL-2 inhibitor, has proven to be quite variable and independent from BCL-2 expression levels (16). Venetoclax showed favorable safety profile and promising results in the setting of relapsed/refractory disease (17) and there are plans to test its use in non-DS patients in combination with first-line chemotherapy in pediatric T-ALL. Moreover, our patient carried activating mutations in *NOTCH1* and *FBXW7* genes, opening the question whether a personalized therapy with NOTCH1 inhibitors might be indicated. Indeed, NOTCH1 inhibitors have been investigated in preclinical models and early phase clinical trials and could represent a further resource for treatment of T-ALL in DS patients (18).

## Conclusion

This case report provides the first detailed genetic characterization of T-ALL in a child with DS. The finding that our patient's T-ALL harbors key somatic mutations typical of T-ALL, specifically, *NOTCH1* and *FBXW7* variants, suggests a sporadic leukemogenesis pathway rather than the DS-specific pathogenesis observed in BCP-ALL.

It remains an open question whether the NFAT pathway inhibition, which regulates T-cell development and function and has been observed in children with DS, is a protective mechanism that reduces the incidence of T-ALL, or if the rare cases that do arise, like ours, have acquired specific non-DS-related driver mutations that overcome this inhibition (19). An improved knowledge and characterization of DS T-ALL might be helpful for the treatment of this very rare disease and to establish if the observed molecular findings are characteristic of DS T-ALL patients or reflect individual variability.

In conclusion, also understanding the limited impact of a single case report, we confirm the rarity of T-ALL in DS patients, the high susceptibility to toxicity secondary to intensive chemotherapy, and that a difficult disease such as T-ALL can be approached with curative intent also in DS patients. We also consider important to perform a complete evaluation of cytogenetic and molecular characteristics as well as of the treatment response in order to better calibrate the treatment intensity.

A global effort, such as a Ponte di Legno initiative (4), could be worth gathering wider information from several international cooperative groups on such extremely rare patients and possibly come up with stronger recommendations on the treatment of such rare, difficult and coexistent conditions.

## Data Availability

The datasets presented in this study can be found in online repositories. The names of the repository/repositories and accession number(s) can be found below: https://www.ebi.ac.uk/metagenomics/,E-MTAB-16715.
